# Role of physical medicine for cancer rehabilitation and return to work under the premise of the “Wiedereingliederungsteilzeitgesetz”

**DOI:** 10.1007/s00508-019-1504-7

**Published:** 2019-05-13

**Authors:** Fadime Cenik, Bruno Mähr, Stefano Palma, Mohammad Keilani, Thomas Nowotny, Richard Crevenna

**Affiliations:** 1 Department of Physical Medicine, Rehabilitation and Occupational Medicine, Medical University of Vienna, Währinger Gürtel 18–20, 1090 Vienna, Austria; 2 Rosalienhof, Bad Tatzmannsdorf, Burgenland Austria

**Keywords:** Workability, Part-time reintegration, Exercise, Experts’ survey, Cancer

## Abstract

**Background:**

With the intention of enabling people a phased return to work after long-term sick leave the so-called “Wiedereingliederungsteilzeitgesetz” (WIETZ) was implemented in Austria on 1 July 2017.

**Methods:**

To explore the overall awareness about the WIETZ and the value of physical modalities together with further supporting measures in return to work of cancer survivors, a survey by using a self-constructed questionnaire was performed in 30 experts 6 months after the WIETZ came into force.

**Results:**

The awareness of Austrian specialists regarding the WIETZ seems to be excellent. Regarding expert opinions, return to work in cancer survivors is notable hampered in workplaces with great physical stress even in times of the WIETZ, whereas for professions in offices and banks it is easier to return to work, with and without WIETZ. The highest impact on return to work seems to be due to exercise, as an intervention of the field of physical medicine and rehabilitation to improve sensorimotor functions and to increase endurance capacity as well as muscular strength.

**Conclusion:**

Early information about cancer rehabilitation and the WIETZ seems to be necessary to facilitate return to work of cancer survivors. Furthermore, exercise interventions seem to be the most important measures from the field of physical medicine and rehabilitation.

## Introduction

Modern cancer care with increasing survival rates puts the focus on return to work in the cancer survivorship. Cancer rehabilitation has a key role in improving patients’ quality of life, functional performance and participation. These factors have a high impact on return to work in cancer survivors by increasing ability to work, social participation and integrating patients into their normal life. Within this framework, patient-centered programs are deployed to help improving health care efficiency, identifying preferences and values of patients and their families and to help prioritizing goal-concordant care to reduce cancer-related symptoms and cancer treatment-related side effects [1–7].

Approximately 35% of all cancer patients are aged between 15 and 65 years [8–10], whereby approximately two thirds survive the disease (in Europe and Austria, [10]) and in further consequence one third of them finally become unemployed [6, 8–11]. Given the fact that ability to work has existential importance, cancer survivors show a high motivation for return to work [6, 8, 9, 11]. Multidisciplinary programs seem to offer a bridge to return to work [6, 8, 9, 11], especially reconditioning therapies, namely exercise interventions, have been shown to be effective methods in cancer rehabilitation [1, 2, 12–14].

With the intention of enabling a phased return to work after long-term sick leave, a new law called the Part-time Reintegration Act (Wiedereingliederungsteilzeitgesetz, WIETZ) was implemented in Austria on 1 July 2017, which could be relevant especially in patients with cancer or mild mental diseases [1, 3, 5, 15]. This law stipulates a step by step approach in return to work by enabling fewer working hours (reduction of 25–50%) for a predefined time but almost equal pay (compensation by social insurance) and under the condition that employers also agree [1, 3, 5, 15]. With the aid of this measure, cancer survivors can return to work earlier or obtain workability in accordance with their physical and psychological conditions.

The aim of this pilot study was to examine the role of physical medicine in cancer rehabilitation and return to work under the premise of the WIETZ by using the methodological approach of an expert survey.

## Methods

This pilot study was a survey with 30 experts from different fields of medicine (occupational medicine, physical medicine and rehabilitation, oncology, cardiology, radiology, sports science, psycho-oncology and nutrition science) and jurisprudence, which was performed to evaluate the role of physical medicine for cancer rehabilitation and return to work under the premise of the WIETZ, and took place as part of a masters thesis (for the field of medicine for organizations) of the corresponding author [16].

Experts were invited to fill in a self-constructed questionnaire, 6 months after the WIETZ came into force (end of December 2017) which consisted of general questions about demographic data and awareness of the WIETZ, and special questions about measures in cancer rehabilitation, return to work and further fields to facilitate workability in cancer survivors before and after the WIETZ. Furthermore, questions were asked about possible and impossible occupations for an optimal return to work after cancer rehabilitation before and after the WIETZ. The experts were asked to rank their answers from the best to the third best and from the unfavorable to third unfavorable, respectively.

### Statistical analysis

Descriptive analysis was performed for demographic data, whereas remaining questions were analyzed quantitatively and qualitatively for the three best ranked and for all answers for each question.

## Results

All invited experts (*N* = 30) agreed participation and the response rate was 100% within 2 weeks. Experts were from different fields of medicine (occupational medicine, physical medicine and rehabilitation, oncology, cardiology, radiology, sports science, psycho-oncology and nutrition science) and jurisprudence. Awareness about the topic of WIETZ was evaluated very well by the experts themselves. The age of the participants was between 29 and 68 years (mean 48.5a ± 10.4). Demographic data are shown in Table 1.

**Table 1:** General data of participating experts (*N* = 30; 48.5 ± 10.4 years)

	Number	%
F	7	23.3
M	23	76.7
*n.* *s.*	*0*	*0*
V.e. in cancer rehab.	22	73.3
No v.e. in cancer rehab.	8	26.7
*n.* *s.*	*0*	*0*
V.e. in physical med.	16	53.3
No v.e. in physical med.	14	46.7
*n.* *s.*	*0*	*0*
V.e. in occupational med.	12	40.0
No v.e. in occupational med.	17	56.7
*n.* *s.*	*1*	*3*
Know about WIETZ	27	90.0
Do not know about WIETZ	3	10.0
*n.* *s.*	*0*	*0*
Awareness of key points about WIETZ	26	86.7
No awareness of key points about WIETZ	4	13.3
*n.* *s.*	*0*	*0*

*F* female, *M* male, *n.* *s.* not specified, *v.e.* vocational experience, *rehab.* rehabilitation, *med.* medicine, *WIETZ* “Wiedereingliederungsteilzeitgesetz”

Experts ranked some of their statements equally (ex equo). Therefore, there are more answers than expected. According to the experts, these are “of equal rank, depending on the indication”, “depending on the tumor type, patients’ status and complaints”.

In Table 2 results from all special questions are presented. According to experts’ opinions, the best supporting therapies to facilitate return to work in cancer patients could be from the field of physical medicine (active measures to increase strength and endurance capacity of cancer patients, such as training, physiotherapy, occupational therapy, etc.) and it’s modalities (questions 1 and 2 in Table 2). An optimal phased return to work could be promoted best by the WIETZ or flexible working hours (question 3 in Table 2), especially for employees working in offices or banks (question 5 in Table 2). Office and bank employees had better opportunities to return to work before the implementation of the WIETZ (question 4 in Table 2). In contrast, employees with hard physical work seem to have great difficulties in returning to work, even after the WIETZ (question 6 in Table 2). Under the premises of the WIETZ, training has the highest impact on return to work of cancer survivors according to question 7 in Table 2. In addition, further physical therapies would be the best additional measures (question 8 in Table 2).

**Table 2:** Qualitative and quantitative analysis of the special questions (*N* = 30) with multiple answers to eight questions

No	Question	Statements (only) for 1st rank (1.)			Statements for rank 1, 2 and 3 (overall)
		1st place	2nd place	3rd place	
1	In your opinion, with which measure do cancer patients get the most out of or rather are facilitated from the entire portfolio of cancer rehabilitation, e.g. *information, psycho-oncology, dietology/nutritional th., PM*^*a*^ for a vocational and social reintegration? Please indicate a ranking of the three most important measures (1 = the best th., 3 = the third best th.)!	PM (*n* = 20): physioth., exercise, individual physical th. according to requirements	Information (9): profound information also about supporting groups, health care management, rehabilitation counselling	Psycho-oncology (*n* = 5)	PM (*n* = 38): ^b^electrotherapy, occupational th., medical massages
2	In your opinion, with which th. do cancer patients get the most out of or rather are facilitated from the field of *PM*^a^ for a vocational and social reintegration? Please indicate a ranking of the three most important therapies (1 = the best th., 3 = the third best th.)!	Exercise (*n* = 21)	Physioth. (*n* = 8)	Occupational th. (*n* = 1)	Exercise (29)
3	What makes currently cancer patients’ *return to work* easier? Please state up to 3 measures (1 = best measure, 3 = third best measure)!	WIETZ or rather changes in working hours (*n* = 9): good preparation for return to work, part-time work, reduction of working hours, legal framework, flexitime	*Equally ranked:*	Cancer rehabilitation (*n* = 4): timely and sufficient	PM (*n* = 18): physioth., exercise, occupational th., supportive and rehabilitative measures, reconditioning, medical massages
			PM (*n* = 5)		
			Information (*n* = 5)		
4	Please name three professions in which you assume that optimal return to work after cancer rehabilitation was best before the implementation of the *WIETZ* (1 = best occupation, 3 = third best occupation)	Office and bank (*n* = 10): office workers, employment with sedentary work, flexible work structuring, administrative activities, accounting clerks, administrative staff, commercial clerk, bankers	Secure professions (*n* = 6): public officers except police etc., politicians	Extensive autonomy (*n* = 4): leaders	Office and bank (*n* = 20)
5	Please name three professions in which you assume that *optimal return to work *after cancer rehabilitation can/could be made possible *by* the implementation of the *WIETZ* (1 = best occupation, 3 = third best occupation)	Office and bank (*n* = 14): office work, administrative activities without time pressure, administrative staff, receptionists, typists, secretaries, bankers, employees with intellectual work	Social work (*n* = 5): doctors, nurses, psychologists	Teachers (*n* = 2)/secure professions (*n* = 2): public officers	Office and bank (*n* = 21)
6	Please name three professions in which you assume that optimal return to work after cancer rehabilitation will remain difficult or even impossible despite the *WIETZ* (1 = worst occupation, 3 = thirst worst occupation)	Hard physical work (*n* = 13): construction workers, mountain farmers, factory workers, furnace workers, shift workers, pieceworkers, unskilled workers, people with unusual individual performance requirements (e.g. professional athletes)	Public transport (*n* = 4): pilots, air hostesses, bus drivers, tram drivers	Self employee (3)	Hard physical work (*n* = 29): ^b^ industrial workers, (fighter) pilots, professional soldiers, firefighters, roofers, doctors on night shifts
7	Now under the premises of the relatively new WIETZ: According to your expert opinion, which physical therapies in cancer rehabilitation are useful or facilitate return to work under the premises of the WIETZ the most? Please indicate a ranking of the 3 most important physical therapies (1 = the best/most effective th., 3 = the third best th.)	Exercise (*n* = 15)	Physioth. (*n* = 10) (incl. biofeedback)	Cancer rehabilitation (*n* = 2): chance for appropriate cancer rehabilitation, gaining physical and psychological resilience	Physioth. (*n* = 21) (incl. biofeedback)
8	According to your expert opinion, *which additional measures* could make return to work even easier for cancer patients? Please state up to 3 measures (1 = best measure, 3 = third best measure)	PM (*n* = 5): adjunctive (also during cancer treatment), physioth., balneoth.	*Equally ranked*:	*Equally ranked:*	Psycho-oncology (*n* = 14): avoidance of existential fears, psycho-oncological care/help/support, advice for free, early treatment if required, supporting resilience, any form of psychological intervention (treatment, relaxation, coaching)
			Cancer rehabilitation (*n* = 4): for free, outpatient, quick allocation	In-house effort (*n* = 3): ergonomics at working place, good working atmosphere, competent occupational physicians	
			Psycho-oncology (*n* = 4): early treatment, supporting resilience	Social services (*n* = 3): like “fit2work”, individual case management, rehabilitation counselling	
			Flexible working hours (*n* = 4): hourly absence	Acceptance (*n* = 3): information of collaborators, positive feedback by collaborators	
			Information (*n* = 4): more information, information campaigns, information about expected reactions of working environment		

*WIETZ* “Wiedereingliederungsteilzeitgesetz”, *PM* physical medicine, *th*. therapy, *physioth*. physiotherapy, *balneoth*. balneotherapy

^a^PM: physical therapies and modalities such as mechanotherapy (namely including exercise and sports therapy, massages, physiotherapy, occupational therapy, ultrasound, shockwave, electrotherapy, thermotherapy, phototherapy, balneotherapy, hydrotherapy, climatotherapy etc.)

^b^In addition to “most rank first”

## Discussion

Cancer survivors seem to have great difficulties in return to work [6, 8–11]. Approximately one third of cancer survivors in employable age are not able to work after cancer diagnosis or treatment [6, 8–11]. Care in multidisciplinary programs as in cancer rehabilitation seems to be a great support for cancer patients, especially in challenging issues such as workability [6, 8, 9, 11].

Cancer rehabilitation is meanwhile an essential and integral part of cancer treatment, not least because of demonstrable effects in reduction of pain and other deficits and increase of functional performance, participation, quality of life, survival rate and workability [1–6]. Workability in cases of return to work facilitates dealing with cancer diseases, restores personal identity and social functioning and improves general health [17, 18]. Thus, cancer rehabilitation is important to fight against the already high unemployment rate in cancer survivors [19–21].

In addition to cancer diagnosis, prognosis and therapy, type and intensity of work and duration of employment [22, 23], the limiting factors/barriers for a successful return to work could be ethnicity/race, education level and socioeconomic status [24–26], lack of adaptation of workplaces to the needs of disabled employees, feeling of being discriminated in the workplace despite high professional qualifications such as manager [20], as well as heavy physical stress, limited opportunities for decision-making and designing in the workplace and fixed-term employment contracts, etc. [27]. Ho et al. showed higher grades of depression, financial loss, fatigue, great physical symptoms and worse general condition in breast cancer patients with reduced workability [28]. By multidisciplinary interventions for reintegration an acceleration of return to work was shown in patients with (musculoskeletal) pain [29].

As a legal framework condition for a phased return to work after sickness absence, the WIETZ was implemented in Austria on 1 July 2017 [15]. Employers, patients and medical professions (as well as other medical stakeholders) need to be aware of the WIETZ for a realistic return to work management and for a well-functioning interface management system. The Department of Physical Medicine, Rehabilitation and Occupational Medicine of the Medical University of Vienna, as part of the establishment of cancer rehabilitation in Austria, had pioneering status alongside and in cooperation with other institutions and since then has focused on central themes in this field [1–7, 30–34]. In doing so, this survey with 30 specialists from the fields of cancer rehabilitation was performed by a self-constructed questionnaire 6 months after implementing the WIETZ in Austria. The key issues were to focus on experts’ awareness and expertise as well as their opinions about implementation of the WIETZ in cancer patients. All invited experts agreed participation and the response rate was 100% within 2 weeks. Given this fact and taking into account the novelty of the law and the knowledge about its key points, specialists’ awareness in return to work seem to be very high. This could be based on correspondent interests, events concerning this matter or the multidisciplinary cooperation.

One of the important interdisciplinary cooperation for cancer survivors is the so-called CCC-platform for side Eeffects management, supportive care and rehabilitation (CCC-SMSCR) at the Comprehensive Cancer Centre (CCC) of the Medical University of Vienna (General Hospital of Vienna, Austria). There, cancer patients dealing with various functional and symptomatic barriers are treated to increase functional capacity and quality of life, to reduce pain and its consequences, and also to give the opportunity to inform and for networking. In addition, workability, employment and return to work aspects are covered to support immediate return to daily living. By this means and depending on needs, objectives and abilities of each patient, a range of modalities and therapeutic options from the field of physical medicine and rehabilitation could be applied in all phases of medical care and cancer rehabilitation by basic directions of the International Classification of Functioning, Disability and Health (ICF) model. The therapeutic media mainly used are exercise (and recommendations for regular physical activity), physiotherapy and occupational therapy as active measures, of which success is highly dependent on patients’ acceptance and adherence, and largely passive measures, such as (lymphatic) massage, electrotherapy (such as transcutaneous electrical nerve stimulation or galvanic baths), therapeutic ultrasound, shock-wave treatment or climatic and balneological treatment (such as hot packs, fango or mud).

With respect to the question of relevance of modules offered in the context of cancer rehabilitation (from the entire portfolio of cancer rehabilitation, question 1), the results of the expert survey clearly indicate that measures taken by physical medicine were considered to be fundamental to reintegrate cancer survivors. The most common best ranked physical measure before and also after implementation of the WIETZ was exercise (to increase strength, endurance capacity, flexibility and coordination). The need for these measures seems to increase even further as the long-term survival of cancer patients increases. In addition, the timely information of patients (before, during and after rehabilitation) about the WIETZ, legal status regarding dismissal/dismissal protection, possibilities for financial support and also the reference to cancer support groups, cancer aid as well as the course and outcome of this chronic illness seem to have high priority in return to work. Further important points to facilitate return to work for cancer survivors may be psycho-oncological care/help/support for, e.g. supporting resilience and motivation, and avoidance of existential fears, and dietology/nutritional therapy. Nevertheless, employees working in offices or banks seem to have best opportunities to return to work, under the premise of the WIETZ and also before implementation of it, whereas employees with hard physical work seem to have great difficulties in return to work, also despite the WIETZ. On the whole, the WIETZ or rather changes in working hours may be highly effective in supporting cancer survivors in returning to work step by step by ensuring more time for rehabilitation or resting time.

### Limitations

This survey was performed by a self-constructed questionnaire. This approach appears to be well-established but could hide structural weakness. On the one hand, the prompt questioning 6 months after implementation of the WIETZ could be a limitation caused by less information about patients, who had experienced an optimal return to work under the premise of the WIETZ but on the other hand, high awareness was shown by the participants despite the novelty of this law. Another limitation could be the selection of the experts, who were directly or indirectly multidisciplinary cooperating partners; however, this limitation could be weak because of the important role of the participants and their comprehensive supervision all over Austria.

## Conclusion

Cancer patients should be informed from the time of cancer diagnosis about cancer rehabilitation together with ways and possibilities of reintegration measures (workability, WIETZ) Furthermore, employed patients, their employers and physicians have to know about the importance of cancer rehabilitation, especially about physical options, such as exercise. Regularly performed exercise seems to have the highest impact on workability and return to work, which is an important issue for cancer patients. As many survivors are able and willing to work following a cancer diagnosis, future research should focus on rehabilitation and work reintegration concepts.
